# Valuation of Ecosystem Services Based on EU Carbon Allowances—Optimal Recovery for a Coal Mining Area

**DOI:** 10.3390/ijerph20010381

**Published:** 2022-12-26

**Authors:** Alicja Krzemień, Juan José Álvarez Fernández, Pedro Riesgo Fernández, Gregorio Fidalgo Valverde, Silverio Garcia-Cortes

**Affiliations:** 1 Department of Extraction Technologies, Rockburst and Risk Assessment, Central Mining Institute, 40166 Katowice, Poland; 2 School of Mining, Energy and Materials Engineering, University of Oviedo, 33004 Oviedo, Spain; 3 Polytechnic School of Mieres, University of Oviedo, 33600 Mieres, Spain

**Keywords:** ecosystem services, valuation, preference programming, EU carbon dioxide emission allowances, coal mining, RECOVERY project

## Abstract

This paper presents a new way of valuing ecosystem services based on the price of EU carbon dioxide emission allowances. Its main advantage is that it facilitates the monetisation of non-provisioning ecosystem services, which is the Achilles heel of current frameworks. The research approach is built on the notion that land rehabilitation and ecological restoration involve trade-offs between ecosystem services. A quantitative assessment (valuation) of these trade-offs is necessary to make sound decisions. However, using different valuation methods to estimate monetary values creates a non-comparability in the valuation process that is difficult to correct. To address this problem, in the first place, the propagation of imprecise preference statements in hierarchical weighting is proposed, avoiding the non-comparability caused by the different current approaches while reducing the effort of preference elicitation. In the second place, to achieve consistency, monetisation of all non-provisioning ecosystem services was carried on the above comparison and the monetary valuation of the attribute with the most direct and market-related valuation possible: carbon sequestration, using the EU Emissions Trading System. A former coal mining area exemplifies the valuation of ecosystem services provided by alternative ecological restoration scenarios. The aim is to estimate their contribution to human well-being, understand the incentives faced by decision makers to manage ecosystems in different ways and assess the values of alternative solutions. An exercise is then carried out to show that the price of EU carbon permits (as of December 2021) after the price escalation that coincides with phase 4 of the allocation of allowances under the EU Emissions Trading System can be estimated by prioritising biodiversity over other ecosystem services.

## 1. Introduction

Ecosystems provide an excellent framework for analysing the links between people and the environment. Ecosystems are “dynamic complexes of communities of plants, animals and micro-organisms, and the non-living environment that interact as a coherent and functional unit” [1].

In recent literature, the links between ecosystems and the economy are often described using the concept of “ecosystem services” or flows of value that human society receives due to the quantity and state of natural capital [2,3,4]. Ecosystem services add an essential dimension to express land rehabilitation and ecological restoration, which is very important from a societal perspective when considering the capacity of ecosystems to provide multiple ecosystem services. The concept of ecosystem services fills the gap between ecosystem science and the practical application of this knowledge in policy and decision-making, linking socio-economic systems and ecosystems through the flow of ecosystem services.

Attempts to assess the value flows of ecosystem services have been ongoing. De Groot et al. [2] presented a typology for valuing the goods and services of ecosystem functions based on their ecological, sociocultural and economic value. Following this line of work, the Economics of Ecosystems and Biodiversity (TEEB) [5] provided a basis for the monetary valuation of ecosystems and biodiversity by assessing their total economic value (TEV). TEV is “the sum of values of all flows of services generated by natural capital, both now and in the future, discounted appropriately”. Valuation methods were classified according to three approaches: market valuation, revealed preference and stated preference. Their aggregation and weighting to obtain the total value were highlighted as an essential issue due to the “weak comparability” of values. Multi-criteria decision analysis (MCDA) was pointed out as a tool that allows multiple values to be integrated after assigning each of them a relative weight. In addition, transparent deliberative processes will, in their view, facilitate the reduction of risk related to the inherent weaknesses of the MCDA [5].

However, the MCDA was developed to determine the best choice based on the scores of the different criteria and the relative weights given to those criteria. Assigning relative weights to other criteria evaluated with varying assessment methods is complicated.

Many authors have addressed the aggregation or comparison of values attributed to ecosystem services. Hein et al. [6] discussed the spatial scales at which ecosystem services are provided and the implications for the different stakeholder values attributed to ecosystem services. According to them, if all values are expressed by comparable monetary indicators (e.g., consumer or producer surplus), they can be summed. If not, they can be compared using the MCDA. Ahlroth and Finnveden [7] stated that weighting is often used to aggregate results and compare alternatives. However, so far, no set consistently uses monetary values based on actual or hypothetical market valuation of environmental degradation and depletion. Gan et al. [8] considered that measuring sustainability is a challenging task and that each weighting and aggregation method has its strengths, weaknesses, and practical situations, being essential to know “when to use what” but remaining unclear which weighting and aggregation methods are more suitable for different situations. They stated that the “one-size-fits-all” approach for weighting and aggregation is inappropriate and proposed a process for choosing the most suitable weighting and aggregation methods.

A review of the weighting and valuation in selected environmental systems analysis tools developed by Ahlroth et al. [9] showed that there is a need for generic sets of weights, as there is a lack of consistent weighting/valuation sets. In many of them, different environmental impacts are valued with other methods, making them incomparable. Moreover, the used values cover just a few effects, limiting the scope of the analysis. Along the same line, Zhao et al. [10] pointed out that environmental benefits are typically evaluated using environmental indicators with different units and implications, so comparisons among various benefits are challenging to perform.

Damigos [11] examined the valuation of environmental impacts in monetary terms employing cost-benefit analysis (CBA) and environmental liability, also known as natural resource damage assessment (NRDA), recognising that the aggregated values may seriously be affected by the assumptions made and the methods used, as well as by theoretical and practical complexities. Sijtsma et al. [12], seeking to better support decision-making on ecosystem services, argued that a careful combination of MCDA and CBA facilitates evaluations of projects involving natural ecosystem services and agriculture changes. Nevertheless, their methodology avoids monetisation, using cardinal/ratio measures.

Wam et al. [13] advocated exploring the valuation of trade-offs without direct pricing or MCDA but within a scheme of monetary exchange protocols. Saarikoski et al. [14] argued that MCDA better values ecosystem services than CBA and linked monetary valuation techniques. They concluded that there is a need for research on hybrid methodologies combining MCDA and monetary valuation. Other authors, such as Spangenberg and Settele [15], question the monetary valuation of non-market goods and propose to focus economics on measuring only real things.

Bagstad et al. [16] compared different decision support tools for valuing ecosystem services. Some of the most widespread public domain models, such as InVEST and ARIES, quantify services and their trade-offs at the landscape scale to support scenario analysis using biophysical units to which per-unit monetary values can be applied. They typically quantify ecosystem services using tables of coefficients for each land cover type derived from field experiments.

Kang et al. [17] stated that despite many ecosystem service valuation studies, calculated values presented wide variations and discrepancies. They divided valuation methods into eight categories, grouped into two types: the equivalent factor method group and the non-equivalent factor method group. Within the non-equivalent factor group or primary data-based approaches, seven categories were included [5,18]: market price method, shadow price method, avoided cost method, replacement cost method, travel cost method, contingent valuation and choice experiment methods and others.

On the other hand, the equivalent factor method refers to ecosystem services calculated based on the relative weight of a particular ecosystem service compared to the standard (equivalent factor per unit area). In the study by Xie et al. [19], the standard was the natural grain output from 1 ha of farmland. However, using a non-provisioning ecosystem service as the standard makes this approach more reasonable. Using a provisioning ecosystem service may not be feasible in certain areas, and using different services will result in various equivalent factors per unit, making the results noncomparable.

The research approach of this paper is based on the notion that land rehabilitation and ecological restoration involve trade-offs between ecosystem services. A quantitative assessment of these trade-offs is necessary to make sound decisions. By quantifying the costs of alternative land rehabilitation and ecological restoration actions and the provision of ecosystem services, it should be possible to determine which options will provide the most significant benefits.

An integrated assessment of multiple ecosystem services based on alternative but as plausible as possible scenarios will allow policy and decision makers to identify and design appropriate optimal strategies [20]. Generating different scenarios is also essential for monetary valuation, as they allow for analysing changes in service provision needed to quantify trade-offs.

This paper will assess the ecosystem services provided by different ecological restoration scenarios using a coal mining area as an example. The aim is to estimate their contribution to human well-being, understand the incentives faced by decision makers to manage ecosystems in different ways and assess the consequences of alternative solutions.

As in the case of most decision support tools for ecosystem service valuation, non-provisioning ecosystem services will be quantified using tables of coefficients for each land cover type derived from field experiments [16]. With this starting point, this paper will explore ecosystem services valuation employing preference programming through approximate ratio comparisons, a development based on the analytic hierarchy process but with substantial practical potential due to the interactivity of its decision support that only requires linear programming to compute the results [21]. This method allows ambiguous preference statements in hierarchical weighting, reducing the preference elicitation effort. Once a reference attribute is selected, the rest of the attributes will be compared relative to the reference attribute. To achieve uniformity, the monetisation of all non-provisioning ecosystem services will be developed based on the monetary valuation of carbon sequestration using the EU Emissions Trading System [22], an approach not possible to find during the literature review designed for this research. This trading system makes the regulatory ecosystem service of carbon sequestration the most direct and market-related valuation of non-provisioning services possible.

This methodology tries to avoid the non-comparability caused by the current approaches. Using different valuation methods for non-provisioning ecosystem services creates a non-comparability in the valuation process that MCDA can hardly correct.

## 2. Materials and Methods

This section describes the study area, its most relevant ecosystems, the alternative scenarios selected for restoration and the ecosystem services that this area will provide and on which the valuation process will be based. Finally, the valuation methodologies used in this research are described.

### 2.1. Study Area Description

The study area in which this research was carried out was the Figaredo Mine in Asturias (Spain), a closed underground coal mine owned by Hulleras del Norte S.A. (HUNOSA), which is currently undergoing a partial restoration process. Following the closure of the mine, the restoration activities have started a partial refurbishment of the waste heaps area, divided into four sectors.

**Figure 1:**
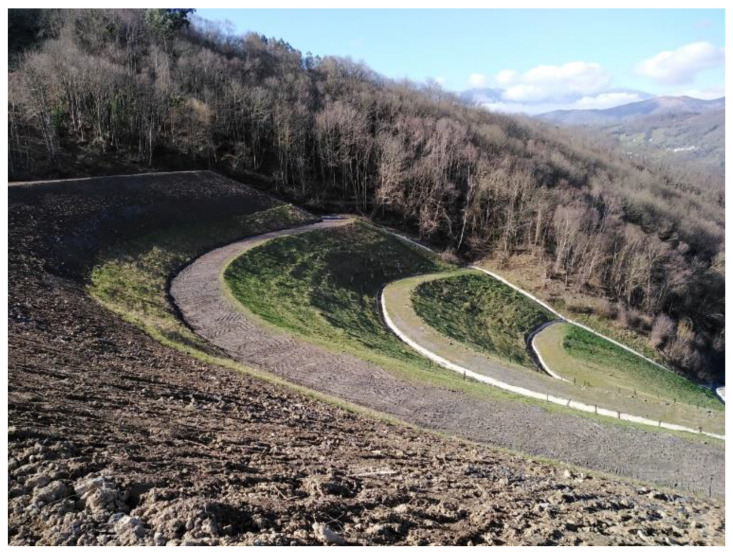
Sector two of the waste heaps area of the Figaredo mine is being restored.

**Figure 2:**
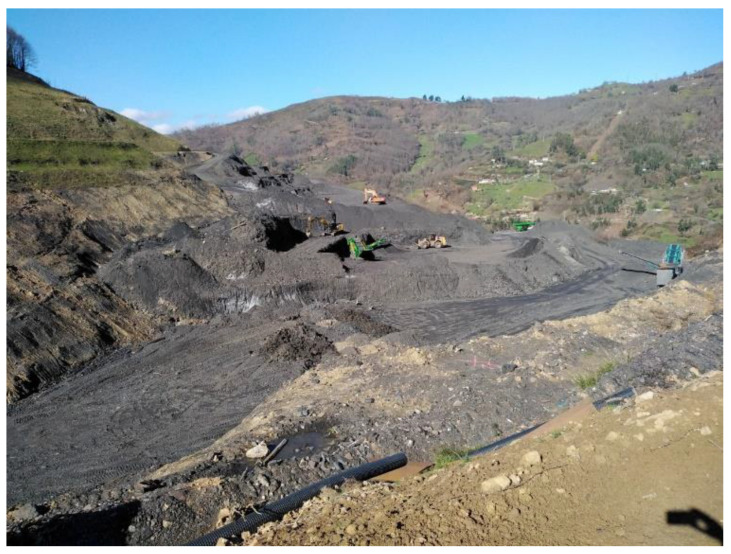
Sector four of the waste heaps area of the Figaredo mine is being re-exploited for coal recovery.

HUNOSA restored sector one in 2009. Sector two is in the process of being restored (Figure 1). Sector four is being re-mined to recover coal (Figure 2), and sector three is being used to store waste from new coal mining in sector four.

The company is also focusing on a new waste heap, as sector three is not large enough to store all the waste produced by the re-exploitation of sector four. The remaining waste heaps cover an area of 67 hectares and are 45 m high. No restoration or rehabilitation has yet been initiated, which provides an excellent opportunity to propose interesting or alternative remediation operations.

The surrounding boundaries of the Figaredo mine area used in the study were defined based on existing spatial connectivity and cohesion. Establishing an ecosystem service context is essential to set appropriate boundaries for the area where the planned activities would lead to changes in land use, property values and ecosystem service potential. For this reason, both the existence of administrative boundaries and the representativeness of the different land covers on the broader area, and the ecosystem services provided by each of them, were taken into account. Finally, given the characteristics of the study area, the boundaries were based on including the whole mine and waste heap area, the whole mountain area up to the top and the valley, including the different villages, the river and several industrial areas. In this way, the selected area could represent the vast territory in which it is embedded.

**Figure 3:**
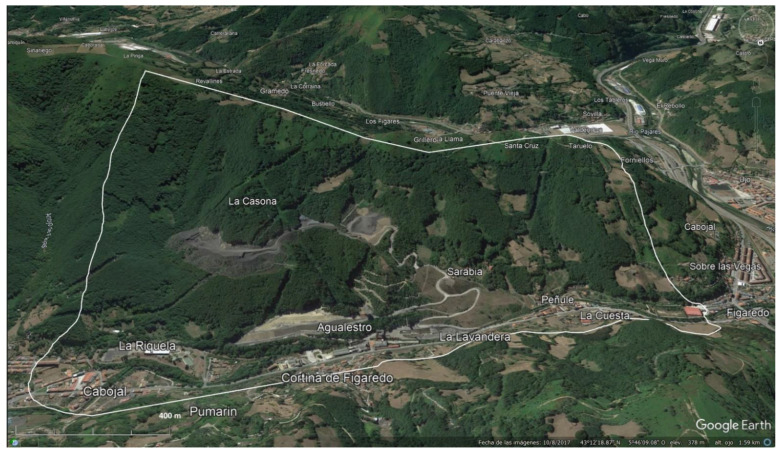
Boundaries of the study area.

The area selected for the Figaredo mine case study is shown in Figure 3, obtained from Google Earth Pro, coordinates 43°12′38.06″ N, 5°46′00.13″ W and mean elevation of 330 m and with an area of 238 ha.

### 2.2. Mapping of Relevant Ecosystems

**Figure 4:**
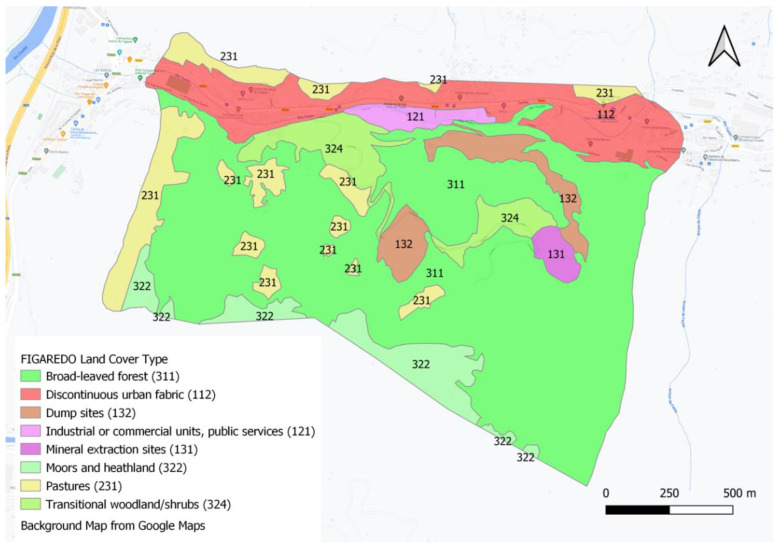
Presentation of the GIS of the CLC classes at Figaredo Mine.

Once the study area was selected, the CLC classes were used to delineate, categorise and map the different ecosystem types in the study area, but a higher resolution was used for field mapping. Figure 4 presents the GIS aspect of CLC classes at Figaredo Mine developed with QGIS 3.8 Zanzibar for the RECOVERY Project [23]. The polygon information available in the GIS is: area (ha), perimeter (km), Level 2 and Level 3 CLC classes and the total area of this specific Level 3 CLC class throughout the case study.

### 2.3. Selection of Restoration Scenarios

For the selection of the alternative scenarios, the characteristics of the Figaredo area have been taken into account, as well as the proposals obtained through a stakeholder consultation within the RECOVERY Project [23]: (1) production of wood as raw material; (2) meat production; (3) broad-leaved forest, similar to those already present in the landscape of the region; (4) land use for renewable energy; (5) self-recolonisation and (6) land use for physical recreation.

**Figure 5:**
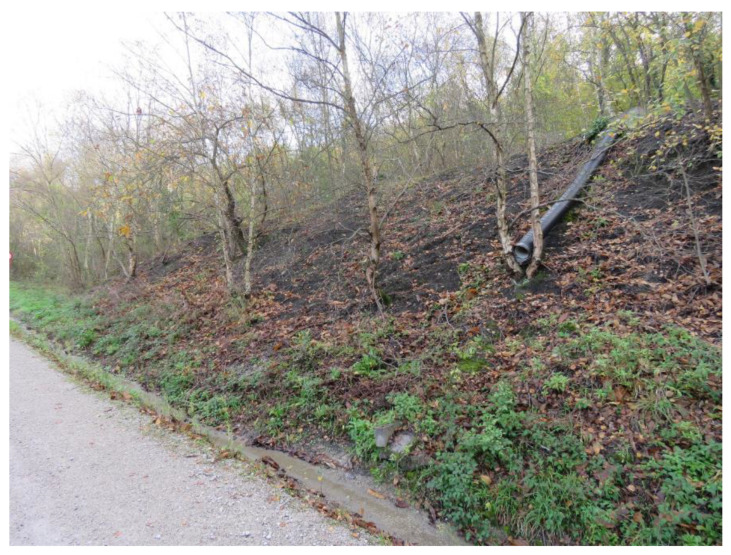
The unrestored area near sector one was mined before 2009.

Three of these scenarios were discarded for different reasons. The land use for renewable energy was not feasible due to the slopes of the waste heaps in the area and the northern orientation. Self-recolonisation was also not advisable, because unrestored areas at the Figaredo mine did not achieve spontaneous revegetation after eleven years without appropriate land soil management (Figure 5). On the other hand, self-generated woody species cannot be compared in terms of productivity and profitability with, for example, pine plantations for timber production, which are widely used in Asturias. Finally, land use for physical recreation was ruled out, as there are numerous recreational facilities related to coal mining in the former coal mining area of Asturias.

Following the re-exploitation of the waste heaps, the first step is to develop slope stability to achieve a suitable final slope configuration. Secondly, hydroseeding must be carried out in each mined area. Both slope stability works and hydroseeding are sunk costs, as in all cases, they have to be incurred and cannot be recovered, and they should not be considered in the cost-benefit assessment.

Based on several trials, an optimal plantation from a forestry perspective was designed with a density of 250 trees/ha in the case of a broad-leaved forest. The species used to reconstruct an Asturian broad-leaved forest stand out for their low mortality. They adapt to all types of terrain, and their soil requirements are much lower than those of other species: *Fraxinus excelsior* (36%), *Betula alba* (36%), *Acer pseudoplatanus* (20%) and *Ilex aquifolium* (8%). In the case of pines, an optimal plantation was designed with a density of 300 trees/ha.

The planting holes have to be sanitised, and topsoil must be added. Then, during the first months after planting, maintenance and watering must be carried out, followed by annual maintenance for at least five years, which includes the following tasks: weeding around each plant for a perimeter of about one metre, hand weeding around the tree, weeding, breaking up large clumps, fertilising with slow-release fertiliser, giving each tree a minimum of 150 g of fertiliser and checking the condition of the stakes. Trees should be planted with a tutor and protective netting. In addition, it is advisable to rinse once a week in the warmer season, with a water supply of about 35 litres per watering plant. The estimated costs calculated by HUNOSA are presented in Table 1.

### 2.4. Selection of Ecosystem Services

An ecosystem services assessment was developed following the baseline mapping of relevant ecosystems. The Common International Classification of Ecosystem Services (CICES) V5.1 [24] was used to achieve a higher degree of standardisation. CICES aims to classify final ecosystem services. These services are final because they are the products of ecosystems (whether natural, semi-natural or highly modified) that directly affect people’s well-being. A key characteristic of final services is that they connect to the underlying functions, processes and structures of the ecosystems that generate them.

Thus, for each relevant land cover, the three main categories of sections (provisioning services, regulating and maintenance services and cultural services), biotic and abiotic, were considered and divided into main types of outputs or processes. Depending on the biological, physical or cultural type or process, these main types were divided into group levels and class categories coded in CICES. Class types within class categories will link ecosystem services to identifiable services, suggesting ways to measure the associated ecosystem services output.

Larondelle and Haase [25] selected eight ecosystem services, indicators and methods for the Mibrag mining sites to value post-mining landscapes using an ecosystem services approach. The ecosystem services were food production, fibre production, freshwater supply, climate regulation, flood regulation, primary production, recreation and biodiversity. Kain et al. [26], in their article on the local consequences of land use alternatives in Stockholm, selected eight ecosystem services based on consultations with ecosystem researchers active in the URBES Project [27]: food supply, energy supply, urban cooling, air quality regulation, carbon sequestration, stormwater retention, physical recreation and mental recreation.

Baró et al. [4] advanced a framework for identifying, mapping and assessing bundles of ecosystem services from a supply and demand approach to inform landscape planning and management and applied it to a metropolitan region. They covered five ecosystem services: food provision, global climate regulation, air purification, erosion control and outdoor recreation. According to Raudsepp-Hearne et al. [3], ecosystem service bundles are “sets of services that occur together repeatedly”. To identify these bundles of ecosystem services, assessments such as the one developed by Burkhard et al. [28], which show the capacity of different land cover types to provide ecosystem services and goods, can be constructive.

Considering this background and the specific features of the study area and the region in which it is located (Asturias, Spain), nine ecosystem services were selected for the Figaredo mining area following the CICES V5.1 classes.

Food and fibre production were considered for provisioning services, and abiotic freshwater supply was not considered. In Asturias, groundwater aquifers are not usually necessary for water supply, both drinking and industrial, as there are many rivers, and water is abundant everywhere.

As for regulating services, climate regulation has been considered in the Figaredo mining area in two ways: through temperature and humidity. According to Schwarz et al. [29], both are complementary indicators to estimate local climate regulation. However, for Laarondelle and Haase [25], the indicator for this service was above-ground carbon storage but as a surrogate for climate regulation at a global scale, not on a local one. In the CICES V5.1 framework, it is referred to as carbon sequestration, as in Kain et al. [26]. It is widely used in all ecosystem service assessments addressing the regulation of the concentration of gases in the atmosphere. Air quality regulation was considered in the Figaredo mine area under air purification, and flood regulation and stormwater retention were considered in water flow regulation. Following Baró et al. [4], erosion control was another ecosystem service considered.

As for cultural services, the biophysical characteristics or qualities of species or ecosystems were considered a good proxy for assessing biodiversity in general and also related to physical and mental recreation.

Finally, net primary production, which represents the net carbon assimilated through photosynthesis by plants and is used to express the net accumulation of carbon by ecosystems, as used by Laarondelle and Haase [25], has not been taken into account, because it has no equivalent in CICES V5.1.

A detailed description of the selected ecosystem services is presented below.

#### 2.4.1. Provisioning Services: Fibre Production

Fibre production through pine plantations to produce wood as raw material is always one of the ecosystem service alternatives traditionally considered in Asturias. The relevant CICES V5.1 code is 1.1.1.2, and the class is “Fibres and other materials from cultivated plants, fungi, algae and bacteria for direct use or processing (excluding genetic material)”. The ecosystem services indicator could be Forest productivity and the quantification method, m^3^/ha/year. A similar one was used by Baró et al. [4] in a study on ecosystem service bundles along the urban-rural gradient, although related to crop production.

#### 2.4.2. Provisioning Services: Food Production

Food supply through cows reared for feed at the Figaredo mine can only occur on pastures. However, horses are raised for feed nowadays, although this is not as common as cows’ cases. The corresponding CICES V5.1 code is 1.1.3.1, and the class “Animals reared for nutritional purposes”. The ecosystem services indicator could be livestock production and the quantification method livestock units/ha/year, as used by Baró et al. [4].

#### 2.4.3. Regulating Services: Climate Regulation (Temperature)

The air temperature was declared as the most apparent/suitable indicator when Schwarz et al. [29] assessed the climate impact of different planning policies in the urban area of Leipzig in Germany, as trees and green regions moderate the climate. The corresponding CICES V5.1 code is 2.2.6.2, and the class “Regulation of temperature and humidity, including ventilation and transpiration”. As air temperature is not easy to estimate spatially, thermal emissions from the Earth’s surface, which indicate the amount of energy emitted by bodies, could be used to measure temperature regulation. In this case, the ecosystem service indicator could be land surface thermal emissions from the Landsat 7 ETM+ satellite (band 6) and the quantification method, the emission index, as used by Schwarz et al. [29].

#### 2.4.4. Regulating Services: Climate Regulation (Humidity)

Humidity (evapotranspiration) was selected by Schwarz et al. [29] as a second indicator for estimating local climate regulation, as forests and green areas influence precipitation and water availability both locally and regionally. Evapotranspiration is the sum of the evaporation of water from the land surface and transpiration from vegetation. As temperature and humidity are not correlated, splitting the two services would facilitate the analysis. In this case, the ecosystem service indicator could be the evapotranspiration potential, as Schwarz et al. [29] used.

#### 2.4.5. Regulating Services: Water Flow Regulation

Water flow regulation is another regulating service, as Asturias is a region with high rainfall. The corresponding CICES V5.1 code is 2.2.1.3 and the class “Hydrological cycle and water flow regulation”. The ecosystem services indicator could be the volume of water retained by vegetation per ha, and the quantification method is the statistical runoff estimated by Nunes et al. [30].

#### 2.4.6. Regulating Services: Erosion Control

Erosion control is also a regulating service to be considered, although its importance in the Asturias region is not very significant. Due to the Asturian climate, with abundant rainfall spread throughout the year and mild temperatures in both winter and summer, vegetation grows very quickly and almost everywhere. The corresponding CICES V5.1 code is 2.2.1.1 and the class “Control of erosion rates”. The ecosystem services indicator could be the soil loss, and the quantification method the soil erosion in g/m^2^ during a monitored period as estimated by Nunes et al. [30].

#### 2.4.7. Regulating Services: Air Purification

Plants provide air purification or removal of air pollution. They have large surface areas for particle deposition and adsorption of gases by the leaf or chemical reactions on the leaf surface. These processes are often referred to as “dry deposition”. The amount of pollution removed by plants depends on their leaves’ size and surface area but can vary depending on climate, time of year and other atmospheric pollutants. The CICES V5.1 code is 2.2.6.1. The class is “Regulation of chemical composition of atmosphere and oceans”. The ecosystem service indicator could be pollutant capture, and the quantification method could be the dry deposition of pollutants, as used by Jones et al. [31].

#### 2.4.8. Regulating Services: Carbon Sequestration

Carbon sequestration was the last regulating service considered. In the case of pastures and coniferous forests, since they are considered provisioning services, this is incompatible with accounting for carbon sequestration as a regulating service. The CICES V5.1 code will be again 2.2.6.1 and the class “Regulation of chemical composition of atmosphere and oceans”. The ecosystem services indicator shall be above-ground carbon storage/ha. The above-ground carbon storage quantification method will be linked to land use in t C/ha, as estimated by Strohbach and Haase [32] in a study on above-ground carbon storage in Leipzig (Germany).

#### 2.4.9. Cultural Services: Qualities of Species or Ecosystems (Biodiversity)

The qualities of species or ecosystems (biodiversity) or biophysical features (landscapes) representing typical Asturian forests (Broad-leaved forests) in the Figaredo Mine area was the last ecosystem service to be analysed. The CICES V5.1 code is 3.2.2.1 and the class “Characteristics or features of living systems that have an existence value”. An example of service should be “areas designated as wilderness”, the ecosystem services indicator could be the type of living systems or environmental settings. The quantification method could be the number of endemic or quasi-endemic species observations. This particular ecosystem service represents an excellent proxy for quantifying biodiversity. Code 3.2.2.2 has the same ecosystem service class and the same indicator. The only difference is that, while the simple descriptor of this code was “things in nature that we want future generations to enjoy or use”, the first code was “the things in nature that we think should be conserved”. In our view, the two are complementary and indissoluble, at least in this case. Although there are different metrics to assess biodiversity considering aspects such as species richness, evenness and identity, for the specific biotope of Figaredo Mine, a study on the nexus between urban shrinkage and ecosystem services by Haase et al. [33] could be used as a reference to simplify the process.

### 2.5. Valuation Methodology

While provisioning ecosystem services will be valued according to market prices, non-provisioning ecosystem services will be quantified before their monetisation, using tables of coefficients for each land cover type derived from field experiments, following Bagstad et al. [16].

The valuation of the provisioning ecosystem services and the costs incurred for any ecosystem services analysed will be done by calculating their net present value (NPV) over a sufficiently long period. A horizon of 70 years or more will be used to consider the residual value equal to zero.

It is then necessary to define the discount rate used in the calculations. Considering such a long horizon and the fact that the average reference rate of the Spanish mortgage market in 2020 is around 2% and the average inflation rate is about 1%, the nominal rate depending on the risk and duration of the investment will be 2%, which is equivalent to a real/constant rate of 1%. Calculations will be made in 2020 real/constant euros, assuming that meat and timber prices will maintain a constant value in 2020 real/constant euros in the coming years. Although by the end of 2021, inflation has increased considerably as a consequence of the effects of the COVID-19 pandemic, it is logical to assume that interest rates should increase by a similar amount, so it would not be wrong to adopt the same 1% as in 2020 as the real rate in the Spanish mortgage market.

On the other hand, in this case, local scaling will be the method selected to transform non-provisioning ecosystem service values into a standard metric, an index between one and ten. Local scaling sets upper and lower bounds using locally measured performance values instead of global scales that may cause irrelevance of differences between local measures. Thus, all criteria performance values will have the same influence on the final scores of the alternatives if they are weighted equally [34], which will not be the case.

To monetise non-provisioning ecosystem services, well-known techniques based on the propagation of imprecise preference statements in hierarchical weighting [35] were used, employing the free software WINPRE—Workbench for INteractive PREference Programming [36].

Local pairwise comparisons with upper and lower limits for criteria and alternatives are made concerning one reference attribute only by introducing imprecise preference statements into value trees. In addition to exact statements, the decision maker can enter interval judgments that indicate ranges for the relative importance of the attributes. Interval judgments for criteria concerning one reference attribute and range-valued information about the outcomes or values of the alternatives are finally processed with linear programming into value intervals and dominance relations.

Hierarchical weighting allows preference statements to be ambiguous, thus reducing the preference elicitation effort. Once a reference attribute—in this case, biodiversity—has been selected, the remaining attributes are compared relative to the reference, taking into account the specific environment and the local scale used.

To achieve consistency, monetisation of all non-provisioning ecosystem services will build on the above comparison and the monetary valuation of the attribute with the most direct and market-related valuation possible: carbon sequestration, which was valuated using the EU Emissions Trading System [22], the world’s first primary carbon market.

## 3. Results

This section will first quantify the ecosystem services provided by the Figaredo mining area. Next, a valuation of these ecosystem services will be made.

### 3.1. Ecosystem Services Quantification

#### 3.1.1. Provisioning Services: Fibre Production

It has not been possible to find a data source to quantify the ecosystem service as the development of pines depends on the specific climate. However, in Asturias pine plantations have, on average, four trees per 10 m^2^, equivalent to 300 trees/ha. After 30–40 years, each pine will produce 2 tonnes of wood with an actual price of EUR 17/tonne.

The source of uncertainty in this valuation will mainly derive from the development of market prices for pine timber as a function of demand/supply and elasticity.

#### 3.1.2. Provisioning Services: Food Production

Again, finding a data source to quantify the ecosystem service was impossible. However, in Asturias 1 ha for feeding cows for meat production can generate around EUR 900 every two years, with EUR 300/year of additional feed costs such as dry grass and feed. The cost of buying a cow ready for insemination is about EUR 1000, plus an insemination cost of EUR 60. The cow will be productive for 14 years.

The source of uncertainty in this valuation will derive from the changing market for beef prices as a function of demand/supply and its elasticity.

#### 3.1.3. Regulating Services: Climate Regulation (Temperature)

The quantification method was the emission index [29] but with the broad-leaved forest as the reference, because its emission value is the lowest. Values were normalised in an index between 1 (highest emission) and 10 (lowest emission), similar to the equation used by Larondelle and Haase [25]:

(1) $$Index\left[i\right]=\left(max_{norm}+min_{norm}\right)-\left[\left(i-min\right)\times \frac{max_{norm}-min_{norm}}{max-min}+min_{norm}\right]$$

The thermal emissivity of the land cover and the respective normalised emission index adapted from Schwarz et al. [29] are presented in Table 2.

Sources of uncertainty in this assessment are the differences in values under different climatic conditions, as these values were obtained for the urban region of Leipzig.

#### 3.1.4. Regulating Services: Climate Regulation (Humidity)

Although there is a linear relationship between evapotranspiration and latent heat of vaporisation (the higher the evapotranspiration, the lower the energy available as sensible heat), this correlation disappears when analysing the total thermal emissivity.

The quantification method will approximate the evapotranspiration potential of the different land cover classes. Schwarz et al. [29] used equations based on empirical estimates and considered soil types and climatic conditions. The evapotranspiration potential f[i] was calculated according to:

f[i] = (max evapotranspiration [i] ÷ ET_0_) (2)

where ET_0_ is the reference evapotranspiration potential of the 12 cm tall grass.

Values were again normalised between 1 (lowest evapotranspiration potential) and 10 (highest evapotranspiration potential). It was unnecessary to reverse the ranking to reflect the lowest evapotranspiration as the highest index, so the following equation was used:

(3) $$Index\left[i\right]=\left[\left(i-min\right)\times \frac{max_{norm}-min_{norm}}{max-min}+min_{norm}\right]$$

The evapotranspiration potential, adapted from Schwarz et al. [29], and the respective normalised emission index are presented in Table 2. Again, sources of uncertainty in this assessment are differences in soil types and values under different climatic conditions, as these values were obtained for the urban region of Leipzig.

#### 3.1.5. Regulating Services: Water Flow Regulation

Some approximations had to be considered, as not all CLC classes of Figaredo mines were presented in Nunes et al. [30]. The values of the rainiest year between the two years analysed (2006) were selected, and the mineral extraction sites and dump sites were assimilated to afforested land. The value chosen for coniferous forests was the mean between broad-leaved forest, and moors and heathland.

According to Tanouchi et al. [37], the range of the impervious surface ratio of the discontinuous urban fabric is between 50% and 80%, so a mean runoff value of 65% of the total rainfall was assigned to both the discontinuous urban fabric and industry or commercial units. The quantification results are presented in Table 2, and a water flow regulation index is calculated according to Equation (1).

The assessment’s sources of uncertainty will be the different values in different climatic environments/conditions and assumptions based only on one year’s rainfall.

#### 3.1.6. Regulating Services: Erosion Control

Using the same assumptions as with water flow regulation and values from the same year (2006) [30], Table 3 presents soil erosion in g/m^2^ and an erosion control index calculated according to Equation (1). In the case of the discontinuous urban fabric and industry or commercial units, as the non-impervious surface, according to Tanouchi et al. [37], was 35%, this percentage was used to calculate their soil erosion based on that of mineral extraction sites and dump sites.

The assessment’s sources of uncertainty will be the different values in different climatic environments/conditions and assumptions based only on one year’s rainfall.

#### 3.1.7. Regulating Services: Air Purification

For reference, the pollutant capture from Jones et al. [31] was used as dry deposition of the following pollutants: sulphur dioxide (SO_2_), coarse particulate matter (PM10), fine particulate matter (PM2.5), ammonia (NH_3_), nitrogen dioxide (NO_2_) and ozone (O_3_). Other interesting studies consider CO, but the variations should not be significant as the pollutants will be considered together.

Table 3 presents the dry deposition of pollutants by land cover classes adapted from Jones et al. [31] and a pollutant dry deposition index calculated according to Equation (3).

Again, sources of uncertainty in the assessment will be the different values in different climatic and geographical environments/conditions.

#### 3.1.8. Regulating Services: Carbon Sequestration

Table 3 presents the above-ground carbon storage per land cover to be considered, adapted from Strohbach and Haase [32], and a carbon storage index calculated, according to Equation (3).

In this case, an indirect monetary valuation of the ecosystem service is possible using the EU Emissions Trading System [22]. Sources of uncertainty in the assessment are the values at different locations, as these values were obtained for Leipzig.

#### 3.1.9. Cultural Services: Qualities of Species or Ecosystems (Biodiversity)

Table 4 presents the impact on the biodiversity of the different land cover cases in the Figaredo Mine area, adapted from Haase et al. [33] and the biodiversity index calculated with Equation (3). According to Cavard et al. [38], different tree species, as in a typical broad-leaved forest in Figaredo Mine (mixed forest), are associated with a more prominent diversity provision than in a case of a single-stand forest of conifer plantations. In addition, as conifer plantations will be used for fibre production, their impact on biodiversity was considered at the same level as pastures. On the other hand, moors and heathland and transitional woodland/shrub have been impacted midway between pastures and broad-leaved forests.

Finally, Table 5 summarises the ecosystem service indicators considered important/relevant in the Figaredo Mine area and their quantification methods.

### 3.2. Ecosystem Services Valuation

To determine the revenues of the three scenarios considered feasible: pine plantations for the production of wood as raw material (Fibre), feeding of cows for beef production (Food) and reconstruction of a broad-leaved forest similar to those already present in the landscape of the region (Landscape), firstly, and according to the costs and payments previously analysed, the NPV of the provisioning ecosystem services will be calculated.

Equation (4) presents the NPV per ha of a pine plantation. The cost of tree planting (300 trees/ha) was estimated at EUR 2040/ha, and the costs of clearing and cleaning, slow-release fertiliser and watering at EUR 780/ha/year, which should take place over the first five years. After 35 years, each pine tree was considered to produce 2 tonnes of timber, which, at a real price of 17 EUR/tonne, represents EUR 10,200/ha. A period of 70 years was used to calculate the NPV in order to allow at least two complete periods of pine trees production. The residual value in year 70 is assumed to be zero. This scenario will be called Fibre.

(4) $$NPV_{Fibre}=-2040-\frac{780}{\left(1+0.01\right)}-\ldots -\frac{780}{{\left(1+0.01\right)}^{5}}+\frac{10.200}{{\left(1+0.01\right)}^{35}}-\frac{2040}{{\left(1+0.01\right)}^{36}}-\ldots +\frac{10,200}{{\left(1+0.01\right)}^{70}}=EUR\;2386$$

Equation (5) presents the NPV per ha of feeding cows for beef production. The cost of buying a cow ready for insemination is about EUR 1000, plus an insemination cost of EUR 60. The cow will be productive for 14 years. The cow will generate in meat (a calf) around EUR 900 every two years, with EUR 300/year of feed costs such as dry grass and feed. The residual value in year 70 is also assumed to be zero. This scenario will be called Food.

(5) $$NPV_{Food}=-1060-\frac{300}{\left(1+0.01\right)}-\frac{\left(900-300\right)}{{\left(1+0.01\right)}^{2}}+\ldots -\frac{\left(600-1060\right)}{{\left(1+0.01\right)}^{14}}+\ldots -\frac{600}{{\left(1+0.01\right)}^{70}}=EUR\;3323$$

Finally, Equation (6) presents the NPV per ha, or the actual cost per ha, of planting a broad-leaved forest with a density of 250 trees/ha. As in the case of the Fibre scenario, clearing and cleaning, slow-release fertiliser and watering should take place over the first five years and at the same price of EUR 780/ha/year (Table 1). This scenario will be called Landscape.

(6) $$NPV_{Landscape}=-1700-\frac{780}{\left(1+0.01\right)}-\frac{780}{{\left(1+0.01\right)}^{2}}-\ldots -\frac{780}{{\left(1+0.01\right)}^{5}}=-EUR\;5486$$

Techniques based on the propagation of imprecise preference statements in hierarchical weighting [21] using the WINPRE program [36] were used to estimate the ecosystem services provision of each proposed scenario.

**Figure 6:**
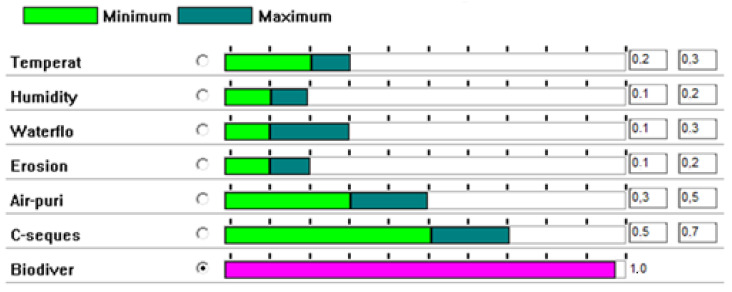
Comparison of attributes (ecosystem services) to the reference attribute (biodiversity).

It was then first necessary to select a reference attribute/ecosystem service. Biodiversity was chosen as the reference attribute, because, of all the attributes, it was the one that allowed comparisons to be made with the others in the most obvious way, which facilitated the development of the process. The rest of the attributes were then compared with the reference attribute using upper and lower limits to allow the existence of imprecise preference statements. Rank orderings should not change with a different anchor, as they are bi-univocal among the various ecosystem services. What may change is only the difficulty of establishing these rank orderings. That is why biodiversity was the anchor selected, as it is the most intuitive among them. Figure 6 presents the results of the comparison carried out using the Delphi method, developed by experts from Hulleras del Norte, S.A. (Spain), the School of Mining, Energy and Materials Engineering of Oviedo (Spain), and the Central Institute of Mining in Katowice (Poland).

While biodiversity was rated as the benchmark with 100% importance, in the case of humidity and erosion, their importance was rated between 10 and 20% of biodiversity. The Asturias region is humid, and erosion is not a problem except for steep slopes. Carbon sequestration was considered between 50 and 70% of biodiversity importance, and so on. No attribute was given more than 100% importance, although this may be the case in other comparisons.

**Figure 7:**
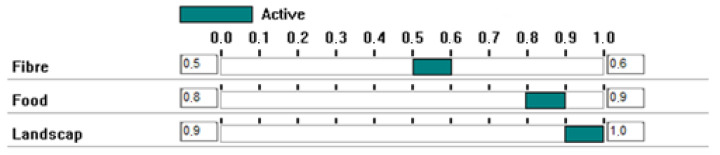
The ranges of values for attribute temperature.

The second step consisted of giving values to the different scenarios/alternatives for each attribute considered. Figure 7 presents the value ranges for the temperature attribute derived from Table 2. These values are derived from the normalised indexes calculated during the ecosystem service quantification. As the computed indices cannot be considered entirely accurate due to the different sources of uncertainty and to reflect these uncertainties in the calculations, when an index is scored with decimals, the selected value range is between the lower and upper integer values of that figure, e.g., the Fibre index was 5.9 (coniferous forest), and the range of values selected is 5–6; the Food index was 8.3 (pastures), and the range of values chosen is 8–9. When an index has an integer value, the range of values selected is between that value and one point less, e.g., the Landscape index is 10 (broad-leaved forest), and the range of values chosen is 9–10.

Following the calculations developed with WINPRE, Table 6 presents the value intervals for the three scenarios considered.

The ecosystem service for which valuation is most feasible must first be selected to monetise the ecosystem services. In this case, the indirect monetary valuation of carbon sequestration through the EU Emissions Trading System (2015) is the most feasible.

According to the EU Emissions Trading System [22], during 2019 and 2020, the period in which this research was developed, the average value of EU Allowances, which allows for the emission of 1 tonne of carbon dioxide equivalent, was about EUR 25/t [39]. As 3.67 t CO_2_ contain 1 t C, the average value of sequestration of 1 t C can be estimated at EUR 91.75/t. Therefore, an above-ground carbon storage rate of 10.0, equivalent to 68.31 t C/ha (Table 3), should be valued at EUR 6267/ha. This value will be the reference value for 100% weighted ecosystem services. An assumption is made that all non-provisioning ecosystem services weighted at 100% are worth the same, given that the specific values for each ecosystem service will come from the relative comparison between them.

Table 7 shows the current valuation of ecosystem services, with biodiversity being the only attribute valued at 100% and used as the reference attribute. Thus, it was given a value of EUR 6267. Finally, the value of the highest possible contribution of ecosystem services in the Figaredo mine area is EUR 17,216/ha.

No discount should be applied to the ecosystem service values in Table 7, as they do not represent real cash flows but timeless values. An example will explain this assertion: the reconstruction of a broadleaved forest, the ecosystem service of climate regulation (temperature), the indicator of land surface thermal emissions and the quantification method of thermal emissivity.

Once the broadleaved forest is planted, and during its growth to maturity, the thermal emissivity will decrease until it reaches a value considered stable. From this point on, the thermal emissivity can be assumed constant and will remain so for as long as the forest survives. Considering that the forest will be maintained over time, the length of its growth period can be regarded as negligible concerning its total duration. Therefore, assuming that the average thermal emissivity is equivalent to its maturity can be considered acceptable.

A somewhat similar explanation would be given in the case of the ecosystem service of carbon sequestration, the indicator of carbon storage and the quantification method of above-ground carbon storage in t/ha. Although the level of carbon storage would increase during the growth of the broadleaved forest, the overall effect on the environment is the ultimate sequestration of a certain amount of carbon. Regardless of whether this total sequestration occurs now or progressively over twenty years, and given that the sequestration will remain the same for many years to come, the overall effect on the environment is the sequestration of this total amount of carbon.

Therefore, if the intention is to value non-provisioning ecosystem services using the current price of EU emission allowances, the most straightforward and practical assumption is to consider their overall impact on the environment at the time of valuation, without applying any financial discount, given their permanence over time.

Finally, Table 8 presents the total values of the different scenarios per ha. To obtain these values, first, the ecosystem service values were calculated by multiplying the value of the highest possible contribution of ecosystem services in the Figaredo Mine area that are obtained in Table 7 (EUR 17,216/ha) by the mean of each interval shown in Table 6. Second, the NPVs obtained in Equations (4)–(6) are added to the ecosystem services values, giving the total value of the different scenarios per ha.

As the difference between the fibre and food production scenarios is negligible (only 3.3%), both can bring similar value to society in the case of Figaredo Mine. Therefore, the selection between them should be based on the ease of undertaking, measured in the lower investment needed to realise the scenario. Food production should then be selected for the specific case of Figaredo Mine.

## 4. Discussion

**Figure 8:**
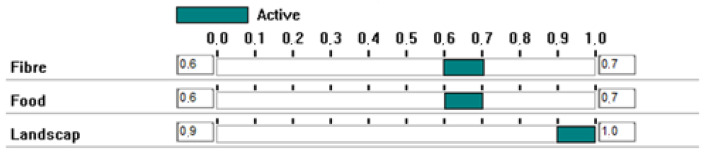
The ranges of values for attribute biodiversity.

An exercise was carried out to estimate what the price of EU allowances would have to be for the Landscape scenario to be chosen. The Landscape scenario is the one that prioritises biodiversity, as shown in Figure 8, where the ranges of values for this attribute are presented. This is tantamount to allowing nature (biodiversity) to set the price of EU allowances in the Figaredo Mine environment.

For this purpose, and so that there can be no doubt about the preponderance between the different scenarios, it will be assumed that the value of the Landscape scenario should be at least 25% above the highest value of the other two scenarios, using the same percentage that Harmsworth and Jacoby [40] proposed as the minimum improvement when considering the benefits from change initiatives related with the success of new products. To achieve this goal, the total value of the Landscape scenario should be EUR 38,754/ha, as presented in Table 9.

By dividing the value of ecosystem services in the Landscape scenario by 0.93, it is possible to obtain the updated total value of the highest potential contribution of ecosystem services in the Figaredo Mine area: EUR 47,570/ha. To achieve this result, it is necessary to value 68.31 t C/ha (equivalent to an above-ground carbon storage rate of 10.0) at EUR 17,298. This would mean that the average sequestration value of 1 t C should be estimated at EUR 253.23, divided by the 3.67 t CO_2_ contained in 1 t C, resulting in 1 tonne of carbon dioxide emission equivalent valued at about EUR 69 instead of EUR 25.

This value of EUR 69 is very similar to the price of EU carbon permits on 17 December 2021, EUR 73.5 [41], after the price escalation that coincides with phase 4 of the allocation of allowances under the EU Emissions Trading System [22].

## 5. Conclusions

This paper presents a new methodology for valuing ecosystem services based on the price of EU carbon dioxide emission allowances. Its main advantage is that it facilitates the monetisation of non-provisioning ecosystem services, which is the Achilles heel of current frameworks. The main conclusions achieved during the research are presented hereafter.

First, although attempts to assess the value of ecosystem services have been ongoing, their aggregation and weighting to obtain the total value is still highlighted as an essential issue due to the “weak comparability” of values. Assigning relative weights to different criteria evaluated with varying assessment methods is complicated, and calculated values present wide variations and discrepancies.

Second, the methodology presented in this paper can avoid the weak comparability of non-provisioning ecosystem services values by (1) quantifying them before their monetisation using tables of coefficients for each land cover type derived from field experiments, (2) selecting a reference ecosystem service and comparing the rest of them to this reference and (3) monetising them based on the valuation of carbon sequestration using the EU Emissions Trading System. This trading system makes the regulatory ecosystem service of carbon sequestration the most direct and market-related valuation possible of all non-provisioning services.

Third, the reference ecosystem service that allows comparisons to be made with the others in the most obvious or intuitive way is biodiversity, facilitating the development of the valuation process.

Fourth, no discount should be applied to the non-provisioning ecosystem services, as they do not represent real cash flows but timeless values. Meanwhile, provisioning ecosystem services should be valued by calculating their net present value according to market prices. Doing it this way, the results obtained for the different proposed scenarios were very reasonable and in line with what was expected for the study region. Moreover, they had the same orders of magnitude. Therefore, they were comparable, giving confidence that the whole process was going in the right direction

Finally, it was possible to estimate the price of EU allowances after the price escalation that coincides with phase 4 of allowances allocation by prioritising the Landscape scenario. It is tantamount to allowing nature (biodiversity) to set the price of EU allowances in the study area to become the scenario to be chosen.

Phase 4 of allowances allocation makes it necessary to adjust or rethink the valuation process developed. The simplest possibility that could be considered would be to reconsider the importance of biodiversity as a reference attribute compared to other attributes. However, this alternative should be carefully analysed, constituting a fascinating field for future research.

## Figures and Tables

**Table 1:** Tree plantation and maintenance costs.

Item	EUR/m^2^	EUR/ha
Tree plantation (250 trees/ha)	0.170	1700
Clearing and cleaning/year	0.045	450
Slow-release fertiliser/year	0.020	200
Watering/year	0.013	130

**Table 2:** Thermal emissivity, evapotranspiration potential and runoff for the different CLC classes.

CLC Classes	Thermal Emissivity		Evapotranspiration Potential		Runoff	
	Emission	Index	f	Index	% Rainfall	Index
Discontinuous urban fabric (112)	139.4	3.5	0.9	2.8	65.0	1.0
Industry or commercial units (121)	141.5	1.0	0.8	1	65.0	1.0
Mineral extraction sites (131)	137.0	6.4	1.0	4.6	12.3	8.3
Dump sites (132)	139.0	4.0	1.0	4.6	12.3	8.3
Pastures (231)	135.4	8.3	1.1	6.4	0.6	9.9
Broad-leaved forest (311)	134.0	10.0	1.1	6.4	0.1	10.0
Coniferous forest (312)	137.4	5.9	1.3	10	6.2	9.2
Moors and heathland (322)	137.0	6.4	1.1	6.4	12.3	8.3
Transitional woodland/shrub (324)	136.0	7.6	1.1	6.4	0.2	10.0

**Table 3:** Soil erosion, dry deposition of pollutants and above-ground carbon storage for the different CLC classes.

CLC Classes	Soil Erosion		Dry Deposition of Pollutants		Above-Ground Carbon Storage	
	g/m^2^	Index	k/year	Index	t C/ha	Index
Discontinuous urban fabric (112)	193.0	6.9	2.02	1.0	20.0	3.6
Industry or commercial units (121)	193.0	6.9	2.02	1.0	8.52	2.1
Mineral extraction sites (131)	551.3	1.0	2.02	1.0	≈0	1.0
Dump sites (132)	551.3	1.0	2.02	1.0	≈0	1.0
Pastures (231)	2.4	10.0	149.4	6.2	≈0	1.0
Broad-leaved forest (311)	1.4	10.0	258.9	10.0	68.31	10.0
Coniferous forest (312)	15.6	9.6	258.9	10.0	≈0	1.0
Moors and heathland (322)	29.8	9.1	120.2	5.1	4.0	1.5
Transitional woodland/shrub (324)	1.2	10.0	189.6	7.6	10.12	2.3

**Table 4:** Biodiversity impact and respective normalised impact index.

CLC Classes	Impact	Index
Discontinuous urban fabric (112)	0	1
Industry or commercial units (121)	0	1
Mineral extraction sites (131)	1	4
Dump sites (132)	1	4
Pastures (231)	2	7
Broad-leaved forest (311)	3	10
Coniferous forest (312)	2	7
Moors and heathland (322)	2.5	8.5
Transitional woodland/shrub (324)	2.5	8.5

**Table 5:** Summary of ecosystem service indicators and quantification methods used in the Figaredo Mine case study.

Ecosystem Service	Indicator	Quantification Method
Fibre production	Forest productivity	m^3^/ha/year
Food production	Livestock production	units/ha/year
Climate regulation (Temperature)	Land surface thermal emissions	Thermal emissivity
Climate regulation (Humidity)	Evapotranspiration	Evapotranspiration potential
Water flow regulation	Runoff	Runoff in % of total rainfall
Erosion control	Soil loss	Soil erosion in g/m^2^ during a monitored period
Air purification	Pollutant capture	Dry deposition of pollutants in t/year
Carbon sequestration	Carbon storage	Above-ground carbon storage in t/ha
Qualities of species or ecosystems (Biodiversity)	Impact of shrinkage- related cover patterns	Degree of suitability

**Table 6:** Value intervals for the three scenarios considered.

Scenarios	Lower Bound	Mean	Upper Bound
Landscape	0.87	0.93	0.99
Fibre	0.49	0.60	0.71
Food	0.47	0.57	0.67

**Table 7:** The value of the highest possible contribution of ecosystem services per ha.

Attribute/Ecosystem Service	Comparative Average Weight *	Value per ha
Temperature	25%	EUR 1567
Waterflow	20%	EUR 1235
Erosion	15%	EUR 940
Air purification	40%	EUR 2507
Carbon sequestration	60%	EUR 3760
Humidity	15%	EUR 940
Biodiversity	100%	EUR 6267
Total		EUR 17,216

* Comparison of other attributes (ecosystem services) concerning the reference attribute (biodiversity), as presented in Figure 6.

**Table 8:** The total values of the different scenarios per ha.

Scenarios	Highest Ecosystem Service Contribution	Interval Means	Ecosystem Services Values	NPVs	Total Values
Landscape	EUR 17,216	0.93	EUR 16,011	EUR –5486	EUR 10,525
Fibre	EUR 17,216	0.60	EUR 10,330	EUR 2386	EUR 12,716
Food	EUR 17,216	0.57	EUR 9813	EUR 3323	EUR 13,136

**Table 9:** The updated total value of the different scenarios per ha.

Scenarios	Highest Ecosystem Service Contribution	Interval Means	Ecosystem Services Values	NPVs	Total Values
Landscape	EUR 47,570	0.93	EUR 44,240	EUR −5486	EUR 38,754
Fibre	EUR 47,570	0.60	EUR 28,542	EUR 2386	EUR 30,928
Food	EUR 47,570	0.57	EUR 27,115	EUR 3323	EUR 30,438

## Data Availability

The data supporting the reported results can be found at https://recoveryproject.uniovi.es (accessed on 22 May 2021).
